# Spirochetes isolated from arthropods constitute a novel genus *Entomospira* genus novum within the order Spirochaetales

**DOI:** 10.1038/s41598-020-74033-9

**Published:** 2020-10-13

**Authors:** Lucía Graña-Miraglia, Silvie Sikutova, Marie Vancová, Tomáš Bílý, Volker Fingerle, Andreas Sing, Santiago Castillo-Ramírez, Gabriele Margos, Ivo Rudolf

**Affiliations:** 1 Programa de Genómica Evolutiva, Centro de Ciencias Genómicas, Universidad Nacional Autónoma de México, Apartado Postal 565-A, CP 62210 Cuernavaca, Morelos Mexico; 2 Institute of Vertebrate Biology, V.V.I., Czech Academy of Sciences, Květná 8, 603 65 Brno, Czech Republic; 3 Biology Centre, Institute of Parasitology, Czech Academy of Sciences, Branišovská 31, 370 05 Ceske Budejovice, Czech Republic; 4 National Reference Center for Borreliosis at the Bavarian Health and Food Safety Authority, Veterinärstr. 2, 85764 Oberschleissheim, Germany

**Keywords:** Bacteria, Environmental microbiology, Microbial genetics, Microbiology, Molecular biology, Genetics, Genomics, Sequencing

## Abstract

Spirochetal bacteria were successfully isolated from mosquitoes (*Culex pipiens*, *Aedes cinereus*) in the Czech Republic between 1999 and 2002. Preliminary 16S rRNA phylogenetic sequence analysis showed that these strains differed significantly from other spirochetal genera within the family *Spirochaetaceae* and suggested a novel bacterial genus in this family. To obtain more comprehensive genomic information of these isolates, we used Illumina MiSeq and Oxford Nanopore technologies to sequence four genomes of these spirochetes (BR151, BR149, BR193, BR208). The overall size of the genomes varied between 1.68 and 1.78 Mb; the GC content ranged from 38.5 to 45.8%. Draft genomes were compared to 36 publicly available genomes encompassing eight genera from the class Spirochaetes. A phylogeny generated from orthologous genes across all taxa and the percentage of conserved proteins (POCP) confirmed the genus status of these novel spirochetes. The genus *Entomospira* gen. nov. is proposed with BR151 selected as type species of the genus. For this isolate and the closest related isolate, BR149, we propose the species name *Entomospira culicis* sp. nov. The two other isolates BR208 and BR193 are named *Entomospira nematocera* sp. nov. (BR208) and *Entomospira entomophilus* sp. nov. (BR193). Finally, we discuss their interesting phylogenetic positioning.

## Introduction

The phylum Spirochaetes Garrity and Holt 2001 encompasses motile free-living, host associated and parasitic microorganisms all of which are characterised by their distinct helical morphology. Unique spirochetal strains were isolated between 1999 and 2002 in the Czech Republic^1^ from mosquitoes of the genera *Aedes*, *Culex* and blackflies of the genus *Simulium*. The bacteria were mainly found in aquatic larval stages with prevalence ranging from 7 to 90%, especially in *Culex* and *Simulium*. However, the number of collected adult stages was low in most years in all mosquito genera. For *Culex* mosquitoes a reasonable number of adult females were found in 1999 and 2000 and the prevalence of spirochetes ranged between 2 and 5%.

In this paper we have determined the genome sequences of four spirochetal isolates from mosquitoes (BR151, BR149, BR193, BR208) and re-evaluated their taxonomic position. These isolates were previously provisionally named ‘*Candidatus* Spironema culicis’ based on partial 16S rRNA analysis^1^. Our working hypothesis was that these isolates belong to a new genus that is part of the family Spirochaetaceae Swellengrebel 1907. Analyses of conserved proteins and phylogenies confirmed their relationship amongst each other and to other taxa; and suggest that these isolates form indeed a new genus containing several new species.

## Results

### Genome characteristics of *Entomospira*

The genomes of isolates BR151, BR149, BR193 and BR208 were sequenced using Illumina MiSeq and Oxford Nanopore Technology. Hybrid genome assemblies using SPAdes v. 3.9.1^2^ revealed a genome typical for spirochetal bacteria consisting of a chromosome and plasmids. Assembly and summary statistics of the genomes are given in Table 1. The genomes sizes of BR149 and BR151 were around 1.77 Mbp with an average mol% G+C of 45.8% (see Table 1 for mol% G+C of plasmids). The chromosomes were 1.6 Mbp in size, and each of the isolates contained two circular plasmids of 98,591 bp and 28,402 bp (Fig. S1). Visualization of the genome assemblies in Bandage^3^ also suggests that the chromosome is circular. Genome annotation conducted in the RAST annotation server^4^ showed that the genomes of BR149 and BR151 contained at least 1687 CDS of which almost 60% encode hypothetical proteins. Two copies of the ribosomal RNAs (16S rRNA (*rrs*), 5S rRNA (*rrf*) and 23S rRNA (*rrl*)) were found. The genome of BR193 had an average size of 1.78 Mb and a mol% G+C of 40.4%. The genome assembled from Nanopore and Illumina data by hybridSPAdes^5^ consisted of a chromosome of 1.49 Mbp and four circular plasmids with sizes of 161,313 bp, 107,998 bp, 22,271 bp and 8241 bp. MetaSPAdes confirmed the presence of four plasmids (with slightly different sizes, see Table 1) whilst plasmidSPAdes^6,7^ confirmed three of the plasmids with sizes of 118,948 bp, 107,988 bp and 22,271 bp (Fig. S1). It is known that plasmidSPAdes may underestimate the number of plasmids actually present in the genome^6^; therefore the information about the plasmids assembled by all three programmes (hybridSPAdes, metaSPAdes and plasmidSPAdes) is given in Table 1. A final judgement about the plasmid content of these genomes will require further study. Annotation of the genome indicated 1707 CDS; 1034 (60%) of which encode hypothetical proteins.

**Table 1:** Assembly statistics and genomic features of four *Entomospira* isolates.

Isolates features	BR149	BR151	BR193	BR208
Host	*Culex pipiens*	*Culex pipiens*	*Aedes cinereus*	*Culex pipiens*
Year of isolation	1999	1999	2000	2001
Partial 16S rRNA gene characterization	Candidatus Spironema culicis	Failed determination	Candidatus Spironema Culicis	Candidatus Spironema Culicis

Assembly statistic	BR149	BR151	BR193	BR208
Genome size (bp)	1,771,032	1,772,169	1,785,838	1,680,798
Number of contigs	8	13	9	7
GC content (%) average	45.8	45.8	40.4	38.5
Shortest contig size	302	302	279	423
Median sequence size	3028	3005	22,271	38,620
Mean sequence size	221,379	136,320	198,426	240,114
Longest contig size	1,639,683	736,781	1,486,042	1,418,720
N50 value	1,639,683	427,344	1,486,042	1,418,720
L50 value	1	2	1	1
Coverage	146	88	104	91
Number of plasmids	2	2	4/3	3
Sizes of plasmids in bp (% G+C)	98,591 (45%)	98,591 (45%)	161,313 (42%)/157,873/118,948^**#**^	132,668 (39.5%)
	28,402 (36%)	28,402 (36%)	107,998 (45.3%)/107,915/107,998	110,286 (34%)
			22,271 (33.6%)/22,199/22,271	21,443 (34%)
			8241 (34%)/8569/missing	

Annotation stats	BR149	BR151	BR193	BR208
CDS	1687	1693	1707	1576
RNAs	47	47	45	46
tRNAs	41	41	41	41
rRNAs	6	6	4	5
Hypothetical proteins	1012	1017	1034	913

ANI values in %	BR149	BR151	BR193	BR208
BR149	100	99	86	86
BR151	99	100	86	86
BR193	86	86	100	84
BR208	86	86	84	100

^#^Plasmid presence and size as determined by hybridSPAdes/metaSPAdes/plasmidSPAdes.

Isolate BR208 has a genome size of 1.68 Mbp and an average mol% G+C of 38.5%. The chromosome was 1.42 Mbp long and it harbors three circular plasmids (132,668 bp; 110,286 bp and 21,443 bp) (Fig. S1). The genome has 1576 CDS of which 58% (913) encode hypothetical proteins. A metal-dependent hydrolase of the beta-lactamase superfamily I was identified in this genome.

### A whole new genus is unveiled

The isolates first characterized as '*Candidatus* Spironema culicis' through partial 16S rRNA sequencing (98–99% nucleotide identity in 690 nucleotides)^1^ were investigated here in depth using draft genomes obtained from Next Generation Sequencing (NGS) via Illumina MiSeq and Nanopore technologies. The adequacy of the 16S rRNA locus for correct assignment of bacterial strains to a species has been questioned and disadvantages of this approach were listed by^8^ including low sensitivity. However, we used 16S rRNA sequences to compare our isolates to related genera in the class Spirochaetes, i.e. *Spirochaeta*, *Alkalispirochaeta*, *Oceanispirochaeta, Leptospira, Treponema, Borrelia*. A phylogeny using all available 16S rRNA sequences of type strains of these and other genera in the class Spirochaetes is shown in Supplementary Fig. S2 (strains included in this analysis are shown in Table S1). Most genera formed well defined cluster in the tree except the genus *Spirochaeta*. The results clearly indicate that our isolates differ significantly from all other genera included. In addition, the sequenced genomes of the four new isolates were compared to a *Spirochaetaceae* genome database, which we created from publicly available genomes in NCBI. This database comprised 36 genomes spanning 8 genera of the 11 listed in the taxonomic classification in NCBI and the List of Prokaryotic Names with Standing in Nomenclature (LPSN)^9^. Genera without completed genome sequences were not included in our study (Supplementary Table S2).

**Figure 1:**
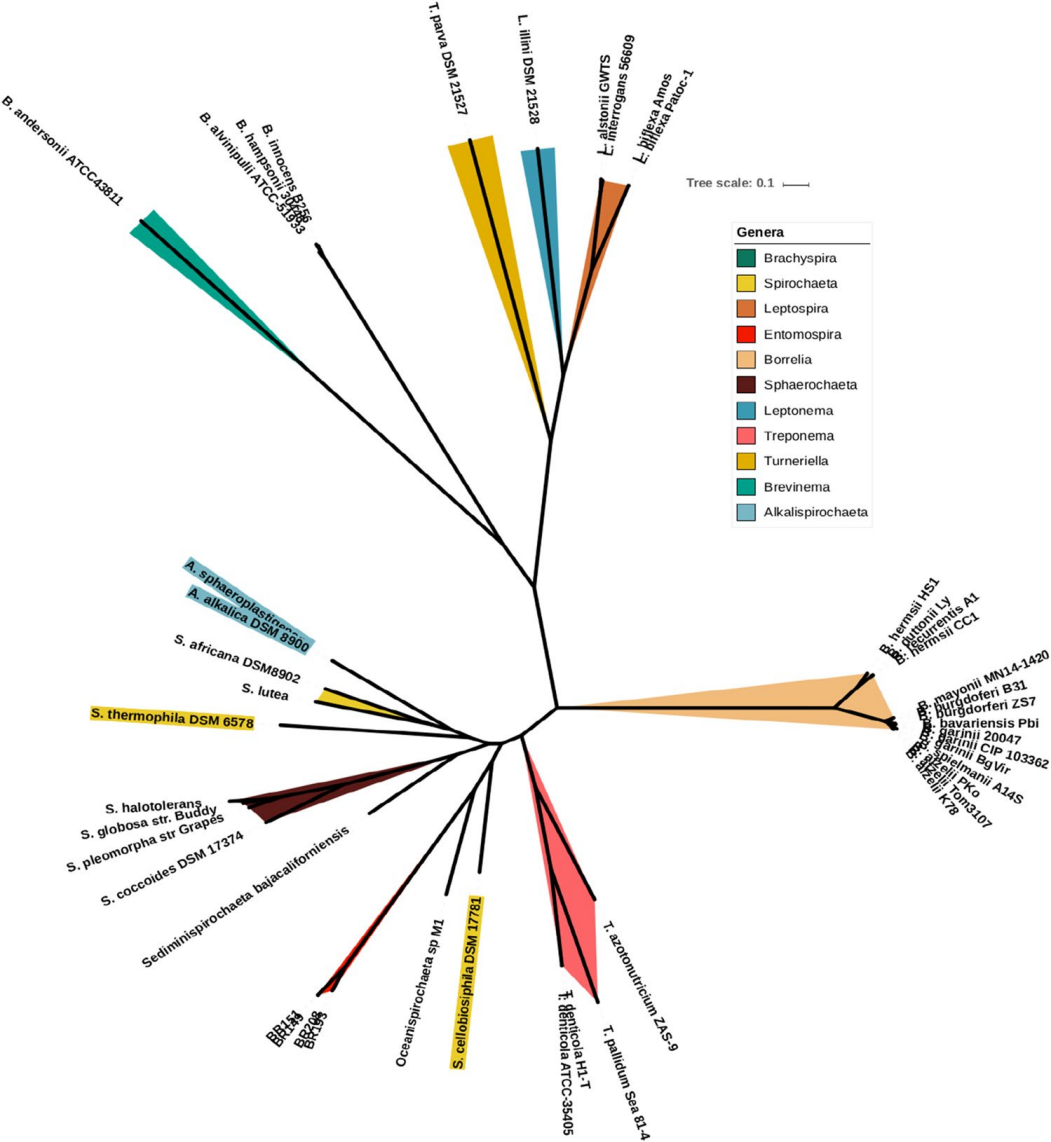
Maximum likelihood phylogenetic reconstruction generated using RaxML v. 8^10^ based on the concatenated alignment prepared in PRANK^11^ of orthologous gene sequences. In addition to our samples, 11 taxa belonging to the class  Spirochaetes were included in the analyses; GenBank accession numbers and further information is given in Table S2. Bootstrap values for the nodes of the tree were above 80%. The scale refers to substitutions per site.

We used orthologous genes (OGs) to carry out a maximum likelihood (ML) phylogenetic reconstruction using RaxML^10^. The resulting tree is shown in Fig. 1, nearly all of the observed nodes were very well supported, showing high (> 80%) bootstrap scores. All previously described genera, except *Spirochaeta*, formed well defined clades, and genera for which only one genome was available clustered as independent lineages. The exception was the species *Spirochaeta cellobiosiphila,* which did not cluster with the other *Spirochaeta* species, and was not part of any other clade. These data suggest that it may be a misclassification even at the genus level.

**Figure 2:**
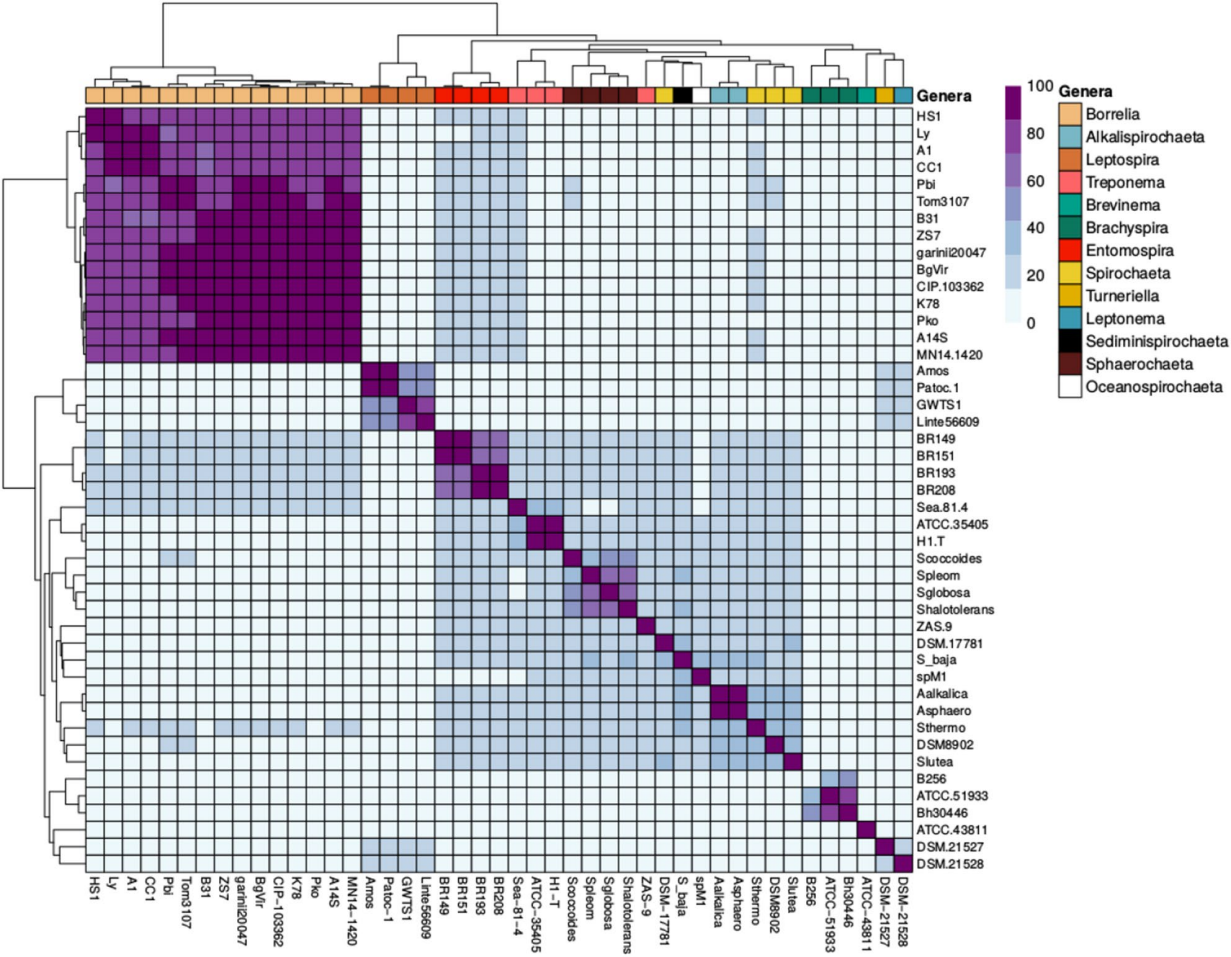
Percentage of conserved proteins (POCP) matrix generated by the method described in^12^. POCP values of species belonging to *Entomospira* gen. nov. are above the genus threshold of 50%, indicating that all strains included belong into one bacterial genus. Of the 12 other genera included some genera such as *Borrelia* were well supported while in other genera some species fall below the 50% threshold. Threshold 0–100% ranges from light blue to purple (right top). Color code for genera are indicate at the right top.

The group of mosquito isolates (BR151, BR149, BR193 and BR208) analysed in this study could not be assigned to any of the previously described species or genera (Figs. 1, S2). We therefore calculated POCP values^12^ by pairwise comparison of all the proteins of the genomes. Values may vary from 0 to 100%, the latter indicating identical protein contents. For two genomes to be considered to belong to the same genus they must have a POCP value of ≥ 50%^12^. Our analysis revealed a different picture for different *Spirochaetaceae* genera. *Borrelia*, *Brachyspira* and the four isolates investigated here formed well defined clusters with POCP values above 50%, suggesting well supported genera (Fig. 2). Also, the genus *Leptospira* is quite well defined, but some of the samples in that group had POCP values slightly below 50%. For some *Leptospira* species, differences in genome size, protein count and/or G+C content were observed but not as pronounced as those observed for *Spirochaeta* and *Treponema* (Supplementary Fig. S3). According to our POCP values, none of the species included here for *Spirochaeta* belongs to same genus, paired comparisons values are well below the cut-off value of 50%. Exactly the same situation is observed with *Treponema*, except for the species *Treponema denticola*, the rest do not seem to belong to the same genus. For our new isolates the evidence was sufficient to affirm that they belong to a new genus with more than one species and we propose the name *Entomospira* genus novum for this group.

**Figure 3:**
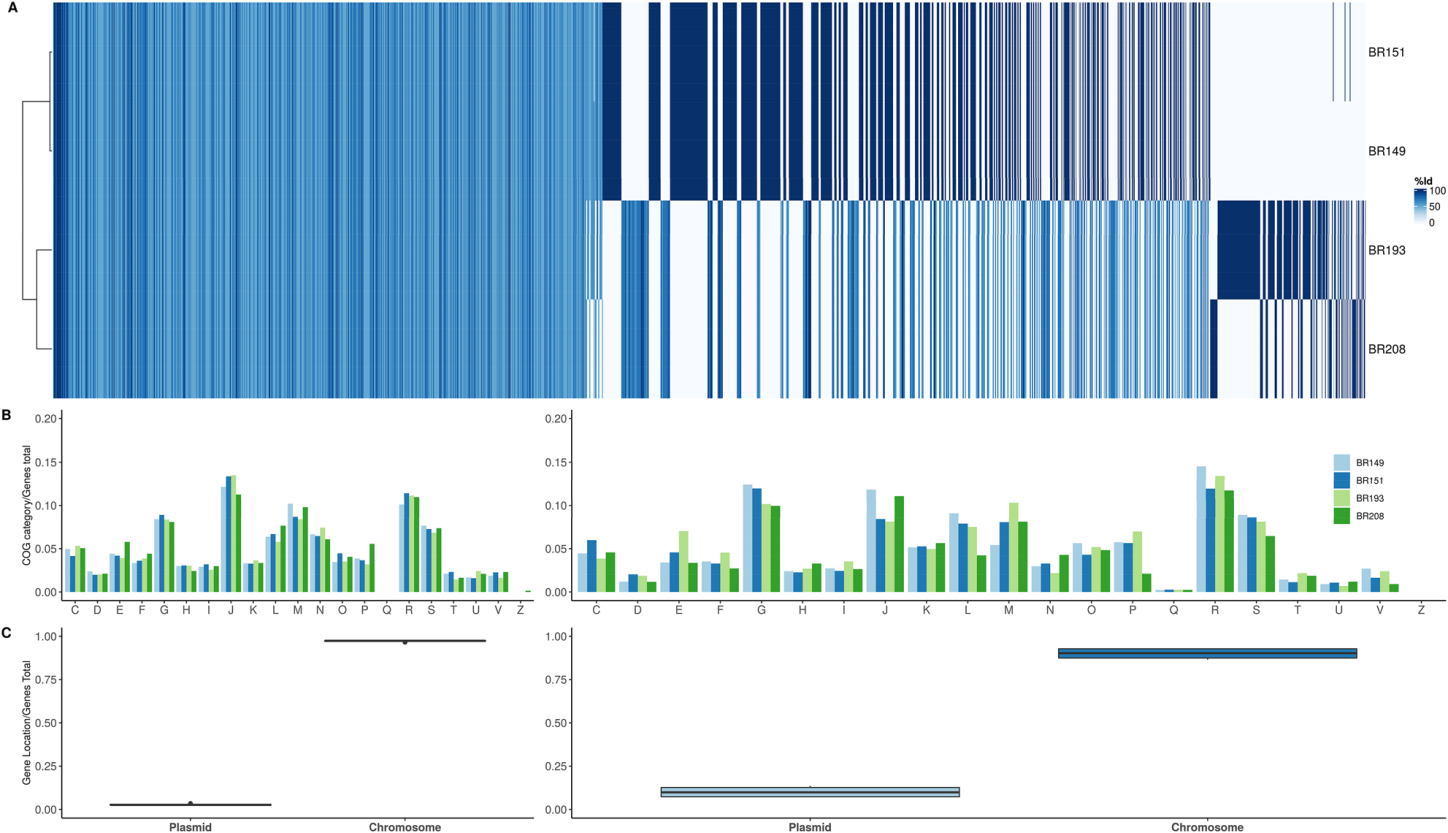
(**A**) Pangenome analysis of *Entomospira* species conducted in Pirate^13^. Shared gene presence per isolate for different percent identity thresholds (50%, 75%, 90%, 95%) were calculated. Gene family presence is indicated by colored columns according to threshold. The scale shows a color gradient for identity thresholds used in the analysis. 97% of the genes shared by all four genomes lie in the range between 50 and 70% identity. The heatmap also shows that shared genes above a 95% identity occur only in BR149 and BR151. (**B**) COG categories^14,15^ identified in the shared genes (left panel) and in the accessory set of genes (right panel). One-letter abbreviations for the functional categories: C, energy production and conversion; D, cell cycle control, cell division, chromosome partitioning; E, amino acid transport and metabolism; F, nucleotide transport and metabolism; G, carbohydrate transport and metabolism; H, coenzyme transport and metabolism; I, lipid transport and metabolism; J, translation, including ribosome structure and biogenesis; K, transcription; L, replication, recombination and repair; M, cell wall structure and biogenesis and outer membrane; N, secretion, motility and chemotaxis; O, molecular chaperones and related functions; P, inorganic ion transport and metabolism; Q, Secondary metabolites biosynthesis, transport, and catabolism; R, general functional prediction only; S, no functional prediction; T, signal transduction; U, intracellular trafficking, secretion, and vesicular transport; V, defense mechanisms. (**C**) For each genome we assessed whether the shared genes are located on the chromosome or on plasmids, shared genes location in the left panel and accessory genes location in right panel.

Within the novel genus two isolates, BR149 and BR151, showed very little divergence in the phylogeny which suggested that they belong to the same species. In addition, their POCP pairwise comparison revealed high values (99.8%). Shared genes between isolates above a 95% protein similarity threshold were significantly more between BR149 and BR151 than between these isolates and BR193 or BR208 or between BR193 and BR208 (Supplementary Fig. S4). Protein identity was also explored in a pangenome analysis that showed that while BR149 and BR151 share a high percentage (94% of annotated CDS) of their proteins at or above a 95% similarity threshold, only a few proteins^13^ are shared between all isolates at this threshold cut-off (Fig. 3A). We compared the distribution of Cluster of Orthologous Genes (COGs), in the proteins shared by the representatives of the genera, and the accessory set of genes (Figs. 3B, S5). More specifically, analyses of functional categories showed that a high number of genes were assigned to the categories “R” (general functional prediction only) and “S” (no functional prediction) (Supplementary Fig. S5) and there were no relevant differences in the distribution of these categories between the shared and accessory genes. Shared and accessory sets of genes are mostly located in the chromosome, with a very small proportion located in plasmids; this is not surprising given that—according to the pangenome analysis—there are big differences in gene content between the three species of the genus that not necessarily reside exclusively in plasmids (Fig. 3C).

Further support that isolates BR149 and BR151 belong to the same species came from results of ANI analysis (Table 1) which confirmed that the two isolates are representatives of the same species, and that the genus so far is composed of two other species represented here by isolates BR193 and BR208.

### Morphology

**Figure 4:**
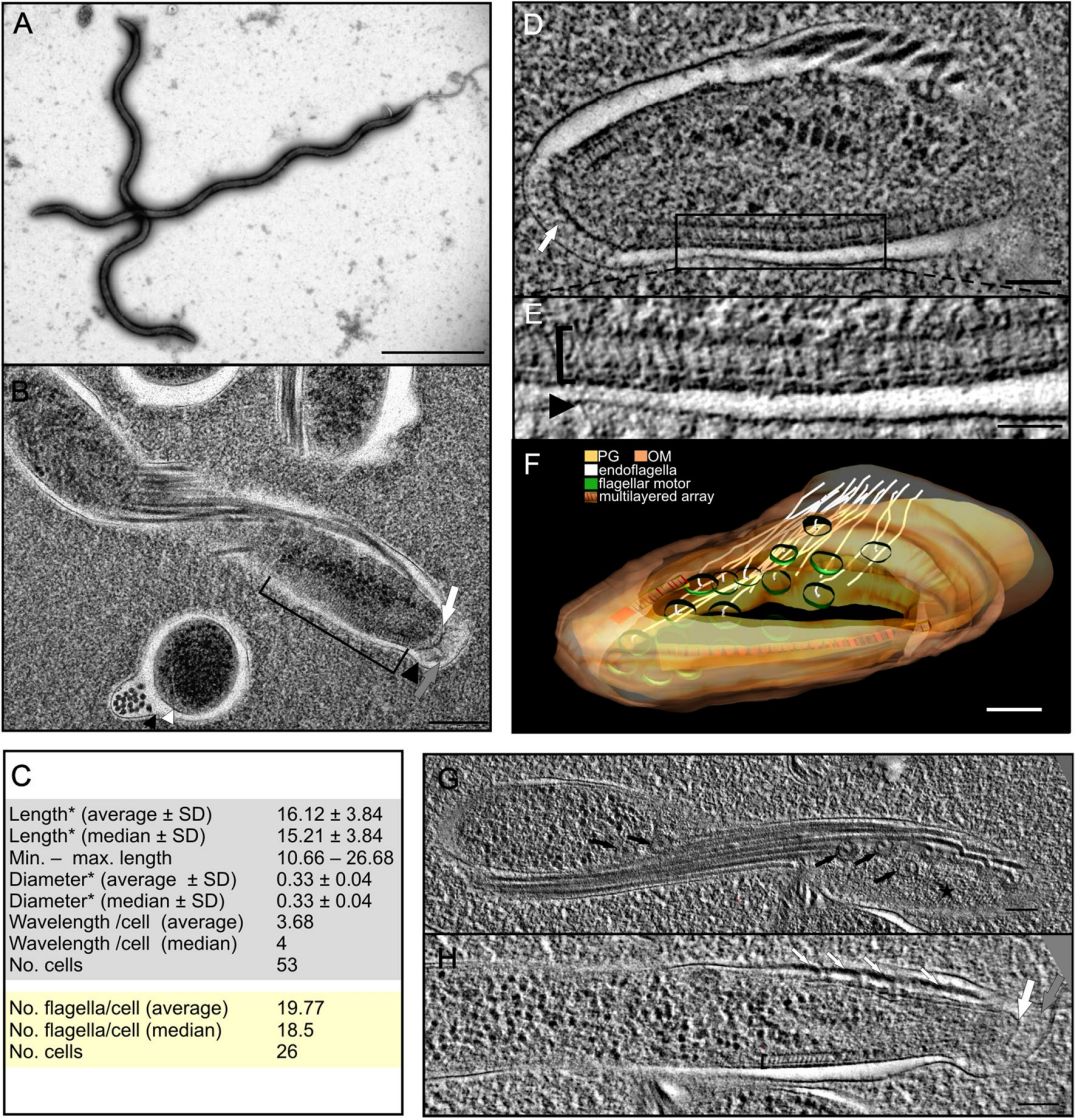
Morphology of *Entomospira* gen. nov. isolate BR151. (**A**) Negatively stained intact bacteria. (**B**) The periplasmic flagella are enclosed in the periplasmic space located between an outer (black arrowheads) and an inner membrane (white arrowhead). Flagellar motors are present near the end of the protoplasmic cylinder and close to approximately 30 nm thick multilayered structure/array (clip). A cap-like structure (white arrow) merges with the inner peptidoglycan layer/cytoplasmic membrane (white arrows) in the cell tip. The terminal part of cells contains fine fibers (grey arrow). (**C**) Quantification of size and morphology of bacteria based on measurements of negatively stained bacteria (light grey box) and cross-sections (yellow box). (**D**) Within the inner membrane, the tomographic slice of the end part reveals the presence of two multilayered arrays located sub-terminally and “cap”-like structure in the tip. Above the cap structure, tiny fibrils (white arrow) filled the cell tip. (**E**) Enlarged area from (**D**). (**F**) 3D reconstruction of the spirochete from (**D**) using the software SerialEM v. 3.7.6^16^ and IMOD v. 4. 10.30^17^ both downloaded from https://bio3d.colorado.edu. (**G,H**) Two tomographic slices from different depths of the same spirochete. At the end part of the spirochete numerous flagellar motors (black arrows) are present with arising flagella (white arrows), tiny filaments (asterisk) and the multilayered array (clip). White arrows show the cap-like structure, grey arrows fibrils at the tip of the spirochete. Bars: 5 µm (**A**), 200 nm (**B**), 100 nm (**D**,**F**–**H**), 50 nm (**E**).

Bacteria of isolate BR151 were 10.7–26.7 µm long and 0.26–0.42 µm in diameter and flat-wave shaped with regular wavelengths (Fig. 4A). The number of wavelengths ranged from 2 to 6. Statistical values for morphological features are listed in Fig. 4C and are compared with other representatives of the family *Spirochaetaceae* in Supplementary Table S3. Periplasmic flagella were sub-terminally attached to both ends of the cell cylinder and arranged in one bundle running in parallel to the long axis of a spirochete (Fig. 4B). We counted between 13 and 32 flagellar filaments on random cross-sections with flagella overlapping in the mid-region. Sub-terminally, multilayered arrays were observed within the peptidoglycan layer/inner membrane (Fig. 4B-clamp,D,E). Flagellar motors were situated next to and below to these arrays (Fig. 4F, Suppl. Movie 1). Lateral areas of the multilayered array contained tiny filaments visible only in appropriately oriented ET slices (Fig. 4G, asterisk). The tip of the cytoplasmic cylinder was closed with a “cap” structure (Fig. 4B,H, white arrow) from which fine fibers expanded into the periplasmic space (Fig. 4B,H, grey arrow).

## Discussion

In this paper, we have determined the genome sequences of four novel spirochete isolates that were obtained from mosquitoes of the genera *Aedes* and *Culex* in the Czech Republic, and have re-assessed their taxonomic status. Comparison of their 16S rRNA sequences to sequences available from genera in the order Spirochaetales and our newly sequenced genomes to 36 publicly available genomes encompassing eight genera from the order Spirochaetales showed that the isolates do not cluster with any previously described genus but form a new genus that belongs to the family Spirochaetaceae. The new genus contains three new species. This conclusion is based on several lines of evidence: (i) the clustering pattern of these samples in phylogenies based on 16S rRNA sequences and on orthologous genes suggests that they are sufficiently divergent from the remaining taxa to be considered a different genus. (ii) POCP values further support the hypothesis that the samples of spirochetes analysed here form a novel genus. (iii) A deeper look into the clade of the new isolates revealed that there are two very similar isolates (BR151 and BR149). This was observed in the phylogenies and with POCP values; their POCP pairwise comparison revealed high values (99.8%) similar to those observed for multiple strains of species in the genus *Borrelia*^18^. These results prompted us to perform an average nucleotide identity (ANI) analysis within the new genus to confirm the relationships between the isolates. ANI is a simple, useful and general descriptor of the genetic relationship between two prokaryotic genomes^19^. In addition, since ANI is based on a large number of genes, it is a better measure of the relationship between strains than data derived from a single gene, such as the 16S rRNA gene^20^. ANI value of > 92% or > 95% have been suggested for isolates that belong to the same species^21^; values found for BR193 and BR208 were lower than this species threshold compared to each other and to the other two isolates (BR149, BR151) strongly supporting the notion that they belong to separate species.

Previous reports described spirochetes that were found in midgut, Malphighian tubules and salivary glands of adults of hematophagous arthropods including mosquitoes, black flies and tabanid flies and many of the described specimen belonged to the genus *Borrelia*^22–29^. However, several strains partially characterized morphologically, physiologically and using 16S rRNA sequences^1,30,31^ were apparently different from the genus *Borrelia*. It is currently unknown which body compartments of insects these spirochetes occupy although some were found in midguts^1^. The latter authors also found that aquatic living mosquito and simuliidae larvae had higher infection rates than either pupae or adults. This is interesting in the light of phylogenetic clustering of these strains. In our study in the phylogeny based on orthologous genes the novel genus clustered as sister clades to spirochete species (*Oceanispirochaeta* sp. M1; *Sediminispirochaeta*) that were described as anaerobic and were associated with coastal marine sediments^32–34^. It would be interesting to investigate the ecology of the novel genus *Entomospira* in more detail and to investigate whether they are commensals or parasites of the arthropods they inhabit. Questions whether these spirochetes may depend on the insect host for nutritional resources, or whether adult insects are simply a way to transport the bacteria to new habitats remain to be answered.

Another observation in our study was that in general the family *Spirochaetaceae* appears to include quite diverse genera composed of strains with high levels of divergence and low POCP values. This had already been observed in other taxa and is likely due to misclassification of strains or to pronounced differences in genomic features^12^. A comprehensive genomic analysis of this family could generate important taxonomic rearrangements as already noted by other authors^32–34^.

Morphologically, bacteria of isolate BR151 had the flat-waved shape that is typical for several other members of *Spirochaetes* such as the Lyme borreliosis group of spirochetes^35^ and the agent of syphilis, *Treponema pallidum*^36^. Other members of the *Spirochaetes* may be more different in their morphologies, e.g. *Leptospira* spp. have a helical cell body with ends that bend either spirally or are hook-shaped^37^. *Brachyspira hampsonii* (swine dysentery) has one to two flat serpentine coils^38^ whereas *Brachyspira hyodysenteriae* has a helical morphology^39^. Another typical feature for all representatives of the *Spirochaetes* is the presence of periplasmic flagella, which reside within a tiny space bordered by the outer membrane/sheat and the peptidoglycan layer associated with the inner cytoplasmic membrane. The number of periplasmic flagella varied in different cells of isolate BR151 from 13 to 32 with a median value of 18.5 and an average of 19.7 (Fig. 4B white and black arrowheads; Fig. 4C). This number is higher than in *B. burgdorferi* (from 7 to 11 endoflagella; Hovind-Hougen 1984), *Treponema* spp. (1–8^40^), *Brachyspira pilosicoli* (8–10^41^); *B. hyodysenteriae* (7–18^42^) and Leptospires, which have a single flagellum attached sub-terminally to each end of the cell without overlapping^43^. This implies that *Entomospira*, if one assumes a similar motility mechanism as in *Borrelia* spirochetes, are likely to have the same or even higher motility abilities, which may be crucial for finding optimal environments.

The cell size of *E. culicis* isolate BR151 is similar to that of other spirochetes. Sub-terminally, an unique multilayered structure is present in the proximity to endoflagella (Fig. 4B-clamp,D,E,H). This structure resembles the chemoreceptor arrays described by cryo-tomography in diverse bacterial species, among others in the cell pole of *Treponema primitia*^44^. Receptor arrays of this type are probably also present in *B. burgdorferi*, but have a different appearance and may be inconsistently positioned^44^. Other authors using cryo-tomography for studying main structural features of Lyme borreliosis spirochetes such as *B. burgdorferi*, *Borrelia afzelii* and *Borrelia garinii* did not mention this structure at all^45^. Finally, the terminus of the isolate investigated here was filled with fine fibers visible in longitudinal section (Fig. 4B,H, grey arrows) and a “cap” merged with the inner peptidoglycan layer/cytoplasmic membrane (Fig. 4B,H, white arrows). Such “cap” structures were revealed by cryo-electron tomography also in *Leptospira* species and *T. pallidum*^46,47^. In *T. primitia*, a cap structure was multi-layered and the fibrils extended from the cells tip above a periplasmic cone^48^.

In conclusion, our genetic, genomic and morphological analyses convincingly show that the isolates investiged here belong to the order Spirochaetales but form a novel and distinct genus. Further investigations are warranted to explore in more detail the ecology of these bacteria.

### Description of Entomospira gen. nov

*Entomospira* (insect inhabiting spiral bacteria). Bacteria with slender, helical-shaped cells, 16.12 μm long (varying from 10.66 to 26.68 μm) and 0.3 μm in diameter. Found preferentially in aquatic larval stages of haematophagous insects belonging to the genera *Culex, Aedes* and *Simulium*. Motility and rotation of cells is conferred by periplasmic flagella overlapping in the middle of the cell. No spores are formed.

The type species for this genus *Entomospira culicis* belongs to the family Spirochaetacea within the order Spirochaetales.

### Description of *Entomospira culicis* sp. nov

*Entomospira culicis* (referring to the mosquito genus from which the type strain was isolated). The main characteristics are as given for the genus. In addition, conditions for growth are 33 °C, pH 7.0 in BSK-H medium. The DNA G+C content of the type strain is 45.7 mol%. It is distinguishable from other species in this genus by ANI values < 92%. The type strain BR151 was isolated from a culicine mosquito (*Cx. pipiens* larvae) in the Czech Republic.

### Description of *Entomospira nematocera* sp. nov

*Entomospira nematocera* (referring to its association with mosquitoes). The main characteristics are as given for the genus. Bacterial growth was observed in BSK-H medium (pH 7.0) at 33 °C. The DNA G+C content of the type strain BR208 is 38.5%. It is distinguishable from other species in this genus by ANI values < 92%. The type strain BR208 was isolated from a mosquito (*Cx. pipiens* larvae) in the Czech Republic.

### Description of *Entomospira entomophilus* sp. nov

*Entomospira entomophilus* (referring to its association with mosquito larvae). The main characteristics are as given for the genus. Growth of bacteria was observed at 33 °C in BSK-H medium, pH 7.0. The DNA G+C content of the type strain BR193 is 40.4%. It is distinguishable from other species in this genus by ANI values < 92%. The type strain BR193 was isolated from a mosquito (*Ae. cinereus* female) in the Czech Republic.

## Experimental procedure

### Isolation, growth, DNA extraction

Isolation of the spirochetes was described in detail by Sikutova et al.^1^. Briefly, using darkfield microscopy spirochete positive mosquito midguts were inoculated in BSK-H medium (Sigma, USA) containing antibiotics (phosphomycin 100 μg/ml and rifampicin 50 μg/ml, Sigma), then incubated at 33 °C and followed for up to six weeks. All isolated strains were subcultured on BSK-H medium without antibiotics and maintained in BSK-H medium with 10% DMSO at – 60 °C for further analyses.

Total DNA was extracted from cultured spirochetes using the Qiagen DNA Mini Kit (Qiagen, Hilden, Germany). DNA concentrations were measured using a Nanodrop spectrometer (Thermo Fisher Scientific, Germany) and an Invitrogen Qubit 3.0 fluorometer (Life Technologies) according to the manufacturer’s recommendations.

### Library preparation and sequencing

Genomes of the four spirochetal isolates BR151, BR149, BR193 and BR208 were sequenced using Illumina MiSeq and Oxford Nanopore Technology. For Illumina sequencing, purified DNA of the isolates was adjusted to 0.2 ng/ul and sequencing libraries were produced using a Nextera XT kit according to the manufacturer’s recommendations (Illumina, Germany). Libraries were quality checked on an Agilent Tape station 2200 and sequenced using a Microreagent kit. Libraries were demultiplexed and trimmed as part of the MiSeq run.

To prepare libraries for Oxford Nanopore Sequencing, the PCR Barcoding kit (SQK-PBK004) was used according to the manufacturer’s recommendations. The resulting libraries were run on a MinION flowcell 9.6 for 16 h. Albacore version 2.0 was used to demultiplex libraries and transfer the sequencing signals into sequences.

### Genome assembly and annotation

The draft genomes of the four isolates, BR149, BR151, BR208 and BR193 were assembled using Spades v3.9.0^2^ for both, Illumina MiSeq and Nanopore. Contigs smaller than 300 base pairs were discarded from analyses. We manually edited our assembly for gap closure and error correction. The quality of the assemblies did not vary substantially, the number of contigs was ≤ 33 in all cases. Coverage was estimated by realigning the reads to the assembly with Bowtie 2^49^, the values were ~ 100 ×. We also run CheckM^50^ to evaluate the quality of the genomes, completeness and contamination values were adequate for taxonomic analysis (more than 95% and less than 5%, respectively).

Nanopore data were assembled together with Illumina MiSeq data using a hybrid assembly strategy in SPAdes v. 3.9.0^2^. The number of contigs was four for BR149 and seven for BR151. Mauve v. 2.3.1^51^. was used to align and compare hybrid assemblies. Readmapping of MiSeq sequences to BR151 revealed a gap in the main chromosome where contigs 3 and 4 were joined. Re-positioning contig 4 to the front of the assembly led to an improvement of gap closure suggesting that the re-shuffled assembly is more correct. Annotation was carried out with PROKKA v1.11^52^ and the Rast annotation server^4^. Proteins were also assigned to functional categories defined in the Cluster of Orthologous Groups database^15^. We used the preformatted RPS-BLAST + database and analyzed the output with a perl script from bac-genomics-scripts^14^. To assess the structure of plasmids, plasmidSPAdes (SPAdes v. 3.14.1) was run^6^. Assembly graphs were visualized in Bandage^3^ and the structure (linearity or circularity) of genome elements assessed.

Sequences have been submitted to NCBI GenBank under Bioproject no. PRJNA611537.

### 16S rRNA phylogeny and orthologous genes search

In Table S1 127 strains (preferentially type strains) are listed from the class Spirochaetes that were downloaded from GenBank and included in a 16S rRNA phylogenetic analysis.

In addition, we downloaded 36 publicly available genomes encompassing eight genera from the class Spirochaetes. There are more than 11 genera described for this class in the NCBI taxonomy database, but whole genome sequences were only available for eight of them. While in some cases there was only one genome sequence available per genus, in other cases like for the species *Treponema denticola* there was a large number of genomes for different isolates. In such cases we choose to include all of them into the analyses. The final data set comprised 36 genomes from eight different genera (Supplementary Table S2).

To identify orthologous genes (OG) for our analyses, we performed a BLASTP search between all the genomes in the data set (all vs all) and used PanOCT^53^ for identification of OG. Since we included isolates of different genera we used a cut-off for amino acid identity (AAI) of 35% and an alignment length cut-off of 60%, for the remaining parameters the default values were used. We found 14 OGs all except for one are ribosomal proteins (Supplementary Table S2). The OGs were aligned in frame using PRANK^11^ and concatenated in order to build a phylogenetic tree with RaxML^10^. The pangenome was also analyzed with Pirate^13^. This software allows exploring several identity thresholds and we used 50, 75, 90, 95% identity thresholds.

### Percentage of conserved proteins

We used a previously reported approach, the Percentage of Conserved Proteins (POCP)^12,18,54^ as another measure of the relatedness between the genomes in our data set. This method was designed to delimit genera. It considers proteins are conserved when they had a BLASTP match with an e-value of less than 1e−05, an amino acid sequence identity of more than 40%, and an aligned region of the query protein larger than 50%. The resulting matrix was visualized with the *ComplexHeatmap* library in R.

We used PIRATE^13^ to analyze the newly described isolates pangenome. PIRATE was run on default settings over a range of amino acid percentage identity thresholds (50, 70, 90, 95).

Shared genes between strains were visualized for 95% similarity thresholds using VennDiagram in R.

### Average nucleotide identity

The average nucleotide identity (ANI) was estimated for the new isolates in order to establish how many species the mosquito associated isolates comprised. ANI was estimated with the *python* module *pyani* using the method ANIm that uses MUMmer (NUCmer) to align the input sequences (https://github.com/widdowquinn/pyani).

### Electron microscopy

Spirochaetes were cultivated at 33 °C in BSK-H Complete medium (Sigma, U.S.A.) Spirochaetes in exponential phases of growth were washed twice in PBS (pH 7.2), and concentrated by centrifugation (8500×*g* for 15 min at 4 °C). The cell pellets were fixed overnight in 2.5% glutaraldehyde in 0.1 M HEPES. After washing in 0.1 M HEPES, bacteria were adhered onto the surface of formvar-carbon coated and glow discharged TEM grids. Grids were washed in H_2_O and stained with 1% aq uranyl acetate for 30 s. The excess of the solution was removed by blotting on filter paper and grids were allowed to dry. For high-pressure freezing-freeze substitution (HPF-FS), pellets were frozen in the presence of 20% bovine serum albumin using a Leica EM PACT2 high-pressure freezer. FS (FSLeica EM ASF2) was carried in 2% OsO_4_ diluted in 100% acetone at − 90 °C for 96 h. Then specimens were warmed up at a rate 5 °C h^−1^, left at − 20°for 24 h and at 4 °C for another 24 h. At room temperature, specimens were rinsed three times in 100% acetone and infiltrated in graded series of SPI-pon resin (SPI) solutions (25%, 50% 75%) diluted in acetone, 1 h at each step. Cells were infiltrated in pure resin overnight, embedded in fresh resin and polymerized at 60 °C for 48 h. Ultrathin sections (70 nm) were cut using an ultramicrotome Leica UCT, counterstained in ethanolic uranyl acetate for 30 min and lead citrate for 20 min. The samples were observed in a transmission electron microscope (TEM) JEOL 2100 F (200 kV, equipped with a high-tilt stage and camera Orius SC 1000, Gatan) and TEM JEOL 1010 (80 kV). For electron tomography (ET), serial ultrathin sections were prepared and carbon coated. Tilt series images were collected at a range of ± 60° with 1° steps with 1,3-pixel size and using SerialEM automated acquisition software v. 3.7.6^16^. Cell volume was reconstructed by joining of three serial tomograms (two dual-axis and one single-axis ET). Images were aligned by means of patch tracking and reconstructed using the IMOD software package v. 4.10.30 (see https://bio3d.colorado.edu/imod/)^17^. The procedures for EM were carried out as described by^55^.

The images for statistical evaluation were taken randomly. Cell lengths and diameters were measured from negatively stained bacteria using ImageJ and results were statistically evaluated. The number of flagella was calculated only from random transverse ultrathin resin sections.

## Supplementary information

Supplementary Information.

Supplementary Movie 1.

## Data Availability

Genome data have been submitted to NCBI GenBank under the Bioproject no. PRJNA611537: Biosample BR151 SAMN14427952; BR149 SAMN14427949; BR193 SAMN14427951; BR208 SAMN14427950.
