# Supplementary material for: Spirochetes isolated from arthropods constitute a novel genus Entomospira genus novum within the order Spirochaetales
Source: Sci Rep. 2020 Oct 13;10:17053. doi: 10.1038/s41598-020-74033-9 (PMC7554043; doi:10.1038/s41598-020-74033-9)
Supplement: Supplementary file 1 — Supplementary Information. [file 41598_2020_74033_MOESM1_ESM.pdf]

Supplementary Information (Graña-Miraglia et al.)

Spirochetes isolated from arthropods constitute a novel genus *Entomospira* genus novum within the order Spirochaetales

Lucía Graña-Miraglia[1#], Silvie Sikutova[2#], Marie Vancová[3], Tomáš Bílý[3], Volker Fingerle[4], Andreas Sing[4], Santiago Castillo-Ramirez[1], Gabriele Margos[4*], Ivo Rudolf[2]

[1]Programa de Genómica Evolutiva, Centro de Ciencias Genómicas, Universidad Nacional Autónoma de México, Apartado Postal 565-A, CP 62210, Cuernavaca, Morelos, México

[2]Institute of Vertebrate Biology, v.v.i. , Czech Academy of Sciences, Květná 8, CZ-603 65 Brno, Czech Republic

[3] Biology Centre, Czech Academy of Sciences, Institute of Parasitology, Branišovská 31, 370 05, České Budějovice, Czech Republic

[4]National Reference Center for Borreliosis at the Bavarian Health and Food Safety Authority, Veterinärstr. 2, 85764 Oberschleissheim, Germany

Supplementary Tables

Table S1 Strains included in 16S rRNA analysis

	GenBank Accession	Genus	Species	Description
AF033305.1	X93927.1	*Alkalispirochaeta*	*A.alkalica*	*Alkalispirochaeta alkalica* DSM 8900 [Z-7491] type 16S rRNA
AJ543428.1	AF373921.1	*Alkalispirochaeta*	*A.americana*	*Alkalispirochaeta americana* ASpG1 type 16S rRNA gene
EU580141.1	HG531387.2	*Alkalispirochaeta*	*A.cellulosivorans*	*Alkalispirochaeta cellulosivorans* JC227 type strain partial 16S rRNA
NR_074168.1	HE806187.1	*Alkalispirochaeta*	*A.sphaeroplastigenens*	*Alkalispirochaeta sphaeroplastigenens* partial 16S rRNA gene type strain JC133T isolate Sp11
AY631880.1	NR_115207.1	*Borrelia*	*B.afzelii*	*Borrelia afzelii* type strain VS461 16S rRNA gene
AY631881.1	NR_116166.1	*Borrelia*	*B.americana*	*Borrelia americana* type strain SCW-41 16S rRNA gene
AY631876.1	NR_118689.1	*Borrelia*	*B.andersonii*	*Borrelia andersonii* strain 21038 16S rRNA gene
AY887899.1	M72397.1	*Borrelia*	*B.anserina*	*Borrelia anserina* 16S rRNA gene
AY796065.1	NR_074854.1	*Borrelia*	*B.bavariensis*	*Borrelia bavariensis* type strain PBi 16S rRNA gene
AY631885.1	NR_148750.1	*Borrelia*	*B.bissettiae*	*Borrelia bissettiae* DN127 type strain 16S rRNA gene
AB721966.1	NC_001318.1	*Borrelia*	*B.burgdorferi sensu stricto*	*Borrelia burgdorferi* B31 type strain 16S rRNA gene
Z21634.1	NR_148824.1	*Borrelia*	*B.californiensis*	*Borrelia californiensis* type strain CA446 16S rRNA gene
Z12817.1	NR_116169.1	*Borrelia*	*B.carolinensis*	*Borrelia carolinensis* type strain SCW-22 16S rRNA gene
AY631895.1	NR_125639.1	*Borrelia*	*B.chilensis*	*Borrelia chilensis* type strain VA1 16S rRNA gene
AB279549.1	NR_025983.1	*Borrelia*	*B.coriaceae*	*Borrelia coriaceae* strain Co53T 16S rRNA gene
EF612284.1	AB113315.1	*Borrelia*	*B.duttonii*	*Borrelia duttonii* 16S rRNA gene
KJ847187.1	NR_043413.1	*Borrelia*	*B.garinii*	*Borrelia garinii* type strain 20047 16S rRNA gene
AY631878.1	NR_102957.1	*Borrelia*	*B.hermsii*	*Borrelia hermsii* DAH 16S rRNA gene
AY631886.1	GU350705.1	*Borrelia*	*B.hispanica*	*Borrelia hispanica* strain CR1 16S rRNA gene
AY293856.1	NR_118688.1	*Borrelia*	*B.japonica*	*Borrelia japonica* type strain HO14 16S rRNA gene
AY631883.1	NR_158115.1	*Borrelia*	*B.lanei*	*Borrelia lanei* type strain CA28-91 16S rRNA gene
AY631888.1	NR_036806.1	*Borrelia*	*B.lusitaniae*	*Borrelia lusitaniae* type strain Poti B2 16S rRNA gene
AY631897.1	NR_152696.1	*Borrelia*	*B.mayonii*	*Borrelia mayonii* type strain MN14-1420 16S rRNA gene
AY631877.1	NR_025861.1	*Borrelia*	*B.miyamotoi*	*Borrelia miyamotoi* type strain HT31 16S rRNA gene
AY631879.1	CP005851.2	*Borrelia*	*B.parkeri*	*Borrelia parkeri* SLO 16S rRNA gene
EF025496.1	AF107367.1	*Borrelia*	*B.recurrentis*	*Borrelia recurrentis* strain A1 16S rRNA gene
AY631882.1	NR_024713.2	*Borrelia*	*B.sinica*	*Borrelia sinica* type strain CMN3 16S rRNA gene
X93928.1	NR_104871.1	*Borrelia*	*B.spielmanii*	*Borrelia spielmanii* type strain PC-Eq17N5 16S rRNA gene
X93927.1	NR_025874.1	*Borrelia*	*B.tanukii*	*Borrelia tanukii* type strain Hk501 16S rRNA gene
AF373921.1	NR_024820.1	*Borrelia*	*B.turcica*	*Borrelia turcica* type strain IST7 16S rRNA gene
HG531387.2	NR_025873.1	*Borrelia*	*B.turdi*	*Borrelia turdi* type strain Ya501 16S rRNA gene
X93926.1	NR_036807.1	*Borrelia*	*B.valaisiana*	*Borrelia valaisiana* type strain VS116 16S rRNA gene
M57740.1	NR_145665.1	*Borrelia*	*B.yangtzensis*	*Borrelia yangtzensis* type strain Okinawa-CW62 16S rRNA
AJ698859.1	Z22781.1	*Brachyspira*	*B.aalborgi*	*Brachyspira aalborgi* type strain NCTC 11492 16S rRNA gene
EU448140.1	EF455559.1	*Brachyspira*	*B.alvinipulli*	*Brachyspira alvinipulli* type strain C1 16S rRNA gene
AJ698092.1	JX232348.1	*Brachyspira*	*B.hampsonii*	*Brachyspira hampsonii* bv. I strain NSH-16 16S rRNA gene
AY995150.1	U14930.1	*Brachyspira*	*B.hyodysenteriae*	*Brachyspira hyodysenteriae* type strain B78 16S rRNA gene
M88722.2	U14920.1	*Brachyspira*	*B.innocens*	*Brachyspira innocens* type strain B256 16S rRNA gene (rrn)
FR749930.1	U23033.1	*Brachyspira*	*B.intermedia*	*Brachyspira intermedia* /Swine intestinal spirochete type strain PWS/A 16S rRNA gene
FR749931.1	CP001959.1	*Brachyspira*	*B.murdochii*	*Brachyspira murdochii* type strain DSM 12563 16S rRNA gene
FR733665.1	AY155458.1	*Brachyspira*	*B.pilosicoli*	*Brachyspira pilosicoli* type strain ATCC 51139 16S rRNA gene
HG965770.2	CVLB01000001.1	*Brachyspira*	*B.suanatina*	*Brachyspira suanatina* type strain AN4859/03 16S rRNA gene
HF968430.2	M59179.1	*Brevinema*	*B.andersonii*	*Brevinema andersonii* type strain ATCC43811 16S rRNA gene
AY337318.1	U42638.1	*Cristispira*	*C.sp.*	*Cristispira* CP1 type strain 16S rRNA gene
		Entomospira	*E. culicis*	*Entomospira culicus* type strain BR151 16 S rRNA gene
		Entomospira	*E. culicis*	*Entomospira culicus* BR149 16 S rRNA gene
		Entomospira	*E. entomophilus*	*Entomospira entomophilus* BR193 16 S rRNA gene
		Entomospira	*E. nematocera*	*Entomospira nematocera* BR208 16 S rRNA gene

	GenBank Accession	Genus	Species	Description
AB598279.1	AY631880.1	*Leptospira*	*L.alexanderi*	*Leptospira alexanderi* serovar Manhao 3 type strain L 60 16S rRNA
KC261846.1	AY631881.1	*Leptospira*	*L.alstonii*	*Leptospira alstonii* genomosp. 1 serovar Sichuan type strain 79601 16S rRNA gene
CP002116.1	AY631876.1	*Leptospira*	*L.biflexa*	*Leptospira biflexa* serovar Patoc type strain Patoc I 16S rRNA
HE806187.1	AY887899.1	*Leptospira*	*L.borgpetersenii*	*Leptospira borgpetersenii* serovar Javanica type strain Veldrat Batavia 46 16S rRNA gene
FR733664.1	AY796065.1	*Leptospira*	*L.broomii*	*Leptospira broomii* type strain 5399 16S rRNA gene
FR749903.1	AY631885.1	*Leptospira*	*L.fainei*	*Leptospira fainei* serovar Hurstbridge type strain BUT 6 16S rRNA
FR749928.1	AY631897.1	*Leptospira*	*L.genomosp.3*	*Leptospira* genomosp. 3 serovar Holland type strain WaZ Holland 16S rRNA gene
LT821384.1	AY631888.1	*Leptospira*	*L.genomosp.4*	*Leptospira* genomosp. 4 serovar Hualin type strain LT 11-33 16S rRNA gene
NR_158116.1	AY631882.1	*Leptospira*	*L.genomosp.5*	*Leptospira* genomosp. 5 serovar Saopaulo type strain Sao Paulo 16S rRNA gene
NR_042797.1	AB721966.1	*Leptospira*	*L.idonii*	*Leptospira idonii* type strain Eri-1 gene for 16S rRNA gene
NR_118718.2	Z21634.1	*Leptospira*	*L.inadai*	*Leptospira inadai* type strain ATCC 43289, partial 16S rRNA
NR_029348.1	Z12817.1	*Leptospira*	*L.interrogans*	*Leptospira interrogans* type strain RGA 16S rRNA gene
U14930.1	AY631895.1	*Leptospira*	*L.kirschneri*	*Leptospira kirschneri* serovar Cynopteri type strain 3522 C 16S rRNA gene
U14920.1	AB279549.1	*Leptospira*	*L.kmetyi*	*Leptospira kmetyi* serovar Malaysia typestrain gene for 16S rRNA
Z22781.1	EF612284.1	*Leptospira*	*L.licerasiae*	*Leptospira licerasiae* serovar Varillal type strain VAR010 16S rRNA
EF455559.1	KJ847187.1	*Leptospira*	*L.mayottensis*	*Leptospira mayottensis* type strain 200901116 16S rRNA gene
JX232348.1	AY631878.1	*Leptospira*	*L.meyeri*	*Leptospira meyeri* serovar Ranarum type strain Iowa City Frog 16S rRNA gene
U23033.1	AY631886.1	*Leptospira*	*L.noguchii*	*Leptospira noguchii* serovar Panama type strain CZ 214 16S rRNA
CP001959.1	AY631883.1	*Leptospira*	*L.santarosai*	*Leptospira santarosai* serovar Shermani type strain LT 821 16S rRNA gene
AY155458.1	AY631877.1	*Leptospira*	*L.weilii*	*Leptospira weilii* serovar Celledoni type strain Celledoni 16S rRNA
CVLB01000001.1	AY631879.1	*Leptospira*	*L.wolbachii*	*Leptospira wolbachii* serovar Codice type strain CDC 16S rRNA
M58795.2	EF025496.1	*Leptospira*	*L.wolffii*	*Leptospira wolffii* serovar Khorat type strain Khorat-H2 16S rRNA
KC665949.1	NR_158116.1	*Marispirochaeta*	*M.aestuarii*	*Marispirochaeta aestuarii* type strain JC444 16S rRNA gene
AF357916.2	LT821384.1	*Oceanispirochaeta*	*O.sediminicola*	*Oceanispirochaeta sediminicola* strain SY2 partial 16S rRNA
NR_029296.1	NR_156083.1	*Pleomorphochaeta*	*P.caudata*	*Pleomorphochaeta caudata* type strain SEBR 4223 16S rRNA
NR_157643.1	AB598280.1	*Pleomorphochaeta*	*P.multiformis*	*Pleomorphochaeta multiformis*/ Sphaerochaeta multiformis 16S rRNA gene
NR_156916.1	NR_156915.1	*Rectinema*	*R.cohabitans*	*Rectinema cohabitans* strain HM 16S rRNA gene
GU562449.1	KC665949.1	*Salinispira*	*S.pacifica*	*Salinispira pacifica* type strain L21-RPul-D2 16S rRNA gene
NR_113327.1	M58795.2	*Saprospira*	*S.grandis*	*Saprospira grandis* type strain LMG 10407 16S rRNA gene
NR_037137.1	KC261846.1	*Sediminispirochaeta*	*S.sinaica*	*Sediminispirochaeta sinaica* type strain SL 16S rRNA gene
NR_113042.1	CP002116.1	*Sediminispirochaeta*	*S.smaragdinae*	*Sediminispirochaeta smaragdinae* type strain 16S rRNA gene DSM 11293 chromosome complete genome
AF033307.1	NR_145842.1	*Sphaerochaeta*	*S.associata*	*Sphaerochaeta associata* strain GLS2 16S rRNA gene
NR_028690.1	AF357916.2	*Sphaerochaeta*	*S.globus*	*Sphaerochaeta globus* type strain Buddy 16S rRNA gene
NR_074169.1	AF357917.2	*Sphaerochaeta*	*S.pleomorpha*	*Sphaerochaeta pleomorpha* str. Grapes 16S rRNA gene
NR_074755.1	X93928.1	*Spirochaeta*	*S.africana*	*Spirochaeta africana* DSM 8902 type 16S rRNA gene
NR_074742.1	X93926.1	*Spirochaeta*	*S.asiatica*	*Spirochaeta asiatica* Z-7591 type 16S rRNA gene
NR_044745.1	M57740.1	*Spirochaeta*	*S.aurantia*	*Spirochaeta aurantia* subsp J1 type 16S rRNA gene
NR_041714.1	AJ698859.1	*Spirochaeta*	*S.bajacaliforniensis*	*Spirochaeta bajacaliforniensis* 16S rRNA gene type strain DSM 16054T
NR_044064.1	EU448140.1	*Spirochaeta*	*S.cellobiosiphila*	*Spirochaeta cellobiosiphila* strain SIP1 type 16S rRNA gene
NR_042486.1	AJ698092.1	*Spirochaeta*	*S.coccoides*	*Spirochaeta* sp. / Sphaerochaeta coccoides SPN1 partial 16S rRNA gene type strain SPN1T
NR_042942.1	AY995150.1	*Spirochaeta*	*S.dissipatitropha*	*Spirochaeta dissipatitropha* type strain ASpC2 16S rRNA gene
NR_025131.1	M88722.2	*Spirochaeta*	*S.halophila*	*Spirochaeta halophila* type strain RS1 16S rRNA gene
NR_026377.1	FR749930.1	*Spirochaeta*	*S.isovalerica*	*Spirochaeta isovalerica* partial 16S rRNA gene type strain DSM 2461T clone 1

	GenBank Accession	Genus	Species	Description
FR749929.1	FR749931.1	*Spirochaeta*	*S.isovalerica*	*Spirochaeta isovalerica* partial 16S rRNA gene type strain DSM 2461T clone 2
NR_156915.1	FR733665.1	*Spirochaeta*	*S.litoralis*	Spirochaeta litoralis partial 16S rRNA gene type strain DSM2029T
NR_156083.1	HG965770.2	*Spirochaeta*	*S.lutea*	*Spirochaeta lutea* partial 16S rRNA gene type strain JC230T
AB598280.1	AY337318.1	*Spirochaeta*	*S.perfilievii*	*Spirochaeta perfilievii* type strain P 16S rRNA gene
NR_145842.1	AB598279.1	*Spirochaeta*	*S.psychrophila*	*Spirochaeta psychrophila* gene for 16S rRNA gene type strain
AF357917.2	HF968430.2	*Spirochaeta*	*S.odontotermitis*	*Spirochaeta* sp. JC202 partial 16S rRNA gene type strain JC202T (Spirochaeta odontotermitis/Alkalispirochaeta odontotermitis)
NR_134803.1	FR733664.1	*Spirochaeta*	*S.stenostrepta*	*Spirochaeta stenostrepta* partial 16S rRNA gene type strain DSM2028T
U42638.1	FR749903.1	*Spirochaeta*	*S.thermophila*	*Spirochaeta thermophila* partial 16S rRNA gene type strain DSM 6578T
NR_042827.1	FR749928.1	*Spirochaeta*	*S.zuelzerae*	*Spirochaeta zuelzerae* partial 16S rRNA gene type strain DSM 1903T clone 1
M59179.1	FR749929.1	*Spirochaeta*	*S.zuelzerae*	*Spirochaeta zuelzerae* partial 16S rRNA gene type strain DSM 1903T clone 2
NR_152696.1	NR_026377.1	*Treponema*	*T.amylovorum*	*Treponema amylovorum* type strain HA2P 16S rRNA gene
NR_148824.1	NR_074168.1	*Treponema*	*T.azotonutricium*	*Treponema azotonutricium* strain ZAS-9 16S rRNA gene
NR_148750.1	NR_042797.1	*Treponema*	*T.berlinense*	*Treponema berlinense* strain 7CPL208 16S rRNA gene
NC_001318.1	NR_029348.1	*Treponema*	*T.brennaborense*	*Treponema brennaborense* type strain DD5/3 16S rRNA gene
NR_145665.1	NR_118718.2	*Treponema*	*T.bryantii*	*Treponema bryantii* type strain RUS-1 16S rRNA gene
NR_125639.1	EU580141.1	*Treponema*	*T.caldarium*	*Treponema caldarium* type strain DSM 7334 16S rRNA gene
NR_025983.1	NR_113327.1	*Treponema*	*T.denticola*	*Treponema denticola* type strain JCM 8153 16S rRNA gene
NR_102957.1	NR_042486.1	*Treponema*	*T.isoptericolens*	*Treponema isoptericolens* strain SPIT5 16S rRNA gene
NR_118689.1	X87139.1	*Treponema*	*T.lecithinolytikum*	*Treponema lecithinolytikum* type strain 16S rRNA gene (patient PFB4G)
NR_118688.1	NR_029296.1	*Treponema*	*T.maltophilum*	*Treponema maltophilum* type strain BR 16S rRNA gene
NR_116169.1	NR_037137.1	*Treponema*	*T.medium*	*Treponema medium* type strain G7201 16S rRNA gene
NR_116166.1	NR_025131.1	*Treponema*	*T.parvum*	*Treponema parvum* type strain OMZ 833 16S rRNA gene
NR_115207.1	GU562449.1	*Treponema*	*T.pectinovorum*	*Treponema pectinovorum* type strain ATCC 33768 16S rRNA
NR_104871.1	NR_044064.1	*Treponema*	*T.pedis*	*Treponema pedis* strain T3552B 16S rRNA gene
NR_074854.1	NR_042942.1	*Treponema*	*T.porcinum*	*Treponema porcinum* strain 14V28 16S rRNA gene
NR_043413.1	NR_074169.1	*Treponema*	*T.primitia*	*Treponema primitia* ZAS-2 16S rRNA gene
NR_024820.1	AJ543428.1	*Treponema*	*T.putidum*	*Treponema putidum* 16S rRNA gene type strain ATCC 700334
NR_025874.1	NR_157643.1	*Treponema*	*T.rectale*	*Treponema rectale* strain CHPA 16S rRNA gene
NR_025873.1	NR_156916.1	*Treponema*	*T.ruminis*	*Treponema ruminis* type strain Ru1 16S rRNA gene
NR_025861.1	NR_044745.1	*Treponema*	*T.saccharophilum*	*Treponema saccharophilum* strain ATCC 43261 16S rRNA gene
NR_036807.1	AF033305.1	*Treponema*	*T.socranskii_buccale*	*Treponema socranskii* subsp. buccale type strain ATCC 35534 16S rRNA gene
NR_036806.1	AF033307.1	*Treponema*	*T.socranskii_paredis*	*Treponema socranskii* subsp. paredis type strain VPI D2B8 16S rRNA gene
AB113315.1	NR_028690.1	*Treponema*	*T.socranskii_socranskii*	*Treponema socranskii* subsp socranskii type strain D56BRIII6 16S rRNA gene
M72397.1	NR_113042.1	*Treponema*	*T.stenostreptum*	*Treponema stenostreptum* strain JCM 16534 16S rRNA gene
AF107367.1	NR_074755.1	*Treponema*	*T.succinifaciens*	*Treponema succinifaciens* DSM 2489 16S rRNA gene
GU350705.1	AY293856.1	*Turneriella*	*T.parva*	*Turneriella parva* serovar Parva type strain H 16S rRNA gene

Table S2 Isolates used for orthologous gene search and analysis of percentage of conserved protein (POCP)

Strain	Species	Genus	Sequence Length	N50	# scaffolds	Protein count	GC(%)	RefSeq assembly accession
DSM 8900	***Alkalispirochaeta alkalica* DSM 8900**	*Alkalispirochaeta*	3,35831	1E+05	71	2766	60,5	GCF_000373545.1
Asphaero	*Alkalispirochaeta sphaeroplastigenens* KCTC15220	*Alkalispirochaeta*	3,35	95244	129	2731	60,5	GCF_002916695.1
K78	*Borrelia afzelii* K78 *	*Borrelia*	1,21928	9E+05	14	1165	27,93	GCF_000962775.1
Pko	*Borrelia afzelii* PKo	*Borrelia*	1,21928	9E+05	18	1165	27,93	GCF_000222835.1
Tom3107	*Borrelia afzelii* Tom3107	*Borrelia*	0,987652	9E+05	3	905	28,16	GCF_000741005.1
Pbi	***Borrelia bavariensis* Pbi**	*Borrelia*	0,986914	9E+05	3	876	28,12	GCF_003814425.1
B31	***Borrelia burgdoferi* B31**	*Borrelia*	1,29508	9E+05	22	1209	28,2	GCF_000008685.2
ZS7	*Borrelia burgdorferi* ZS7	*Borrelia*	1,34549	9E+05	15	1277	28,23	GCF_000021405.1
Ly	*Borrelia duttonii* Ly #	*Borrelia*	1,60791	9E+05	17	1198	27,96	GCF_000019685.1
20047	***Borrelia garinii* 20047**	*Borrelia*	1,229416	9E+05	11	1152	28,1	GCF_003814405.1
BgVir	*Borrelia bavariensis* BgVir	*Borrelia*	0,993811	9E+05	3	882	28,17	GCF_000239475.1
CIP-103362	*Borrelia garinii* CIP 103362	*Borrelia*	1,15611	9E+05	12	1046	28,17	GCF_001922545.1
CC1	*Borrelia hermsii* CC1#	*Borrelia*	1,37793	9E+05	1	1125	30,19	GCF_000956315.1
HS1	*Borrelia hermsii* HS1	*Borrelia*	1,37793	9E+05	12	1125	30,19	GCF_001660005.1
MN14-1420	***Borrelia mayonii* MN14-1420**	*Borrelia*	1,3068	9E+05	16	1126	27,85	GCF_001945665.1
A1	*Borrelia recurrentis* A1 #	*Borrelia*	1,24216	9E+05	8	1029	27,51	GCF_000019705.1
A14S	*Borrelia spielmanii* A14S	*Borrelia*	1,252559	2E+05	49	1118	27,69	GCF_000181895.2
ATCC-51933	***Brachyspira alvinipulli* ATCC-51933**	*Brachyspira*	3,42076	2E+05	22	2968	26,9	GCF_000518245.1
Bh30446	*Brachyspira hampsonii* 30446	*Brachyspira*	3,06547	1E+05	4	2599	27,55	GCF_000316195.1
B256	***Brachyspira innocens* B256**	*Brachyspira*	3,28161	52799	130	2767	27,7	GCF_000384655.1
ATCC-43811	***Brevinema andersonii* ATCC43811**	*Brevinema*	1,5014	65411	39	1439	35,2	GCF_900112165.1
DSM-21528	***Leptonema illini* DSM 21528**	*Leptonema*	4,47525	7E+05	3	4135	54,3	GCF_000243335.1
GWTS1	*Leptospira tipperaryensis* GWTS #	*Leptospira*	4,59189	4E+06	2	4274	42,4	GCF_001729245.1
Ames	*Leptospira biflexa* Ames	*Leptospira*	3,95609	4E+06	3	3691	38,9	GCF_000017605.1
Patoc-1	***Leptospira biflexa* Patoc-1**	*Leptospira*	3,95145	4E+06	3	3695	38,9	GCF_000017685.1
Linte56609	*Leptospira interrogans* 56609 #	*Leptospira*	4,91565	4E+06	5	4057	35	GCF_000941035.1
BR149	*Entomospira culicis*	*Entomospira*	1,769704	2E+06	4	1633	45,77	
BR151	*Entomospira culicis*	*Entomospira*	1,769943	4E+05	7	1636	45,77	
BR193	*Entomospira entomophilus*	*Entomospira*	1,785208	1E+06	7	1683	40,39	
BR208	*Entomospira nematocera*	*Entomospira*	1,680375	1,419	6	1542	38,52	
M1	*Oceanispirochaeta* sp M1 #	*Oceanispirochaeta*	5,88432	1E+05	165	5092	42,9	GCF_003346715.1
Sbaja	*Sediminispirochaeta bajacaliforniensis*	*Sediminispirochaeta*	4,58	2E+05	75	4162	49,2	GCF_000378205.1
DSM-17374	***Sphaerochaeta coccoides* DSM 17374**	*Sphaerochaeta*	2,23	2E+06	1	1846	50,6	GCF_000208385.1

Strain	Species	Genus	Sequence Length	N50	# scaffolds	Protein count	GC(%)	RefSeq assembly accession
Buddy	***Sphaerochaeta globosa* str. Buddy**	*Sphaerochaeta*	3,316	3E+06	1	3028	48,9	GCF_000190435.1
Shalo	*Sphaerochaeta halotolerans* @	*Sphaerochaeta*	2,927	2E+05	49	2663	46,8	GCF_003429665.1
Grapes	***Sphaerochaeta pleomorpha* str Grapes**	*Sphaerochaeta*	3,59	4E+06	1	3192	46,2	GCF_000236685.1
DSM8902	***Spirochaeta africana* DSM8902**	*Spirochaeta*	3,28586	3E+06	1	2781	57,8	GCF_000242595.2
DSM-17781	***Spirochaeta cellobiosiphila* DSM 17781**	*Spirochaeta*	3,94985	2E+05	29	3601	37	GCF_000426705.1
Slutea	***Spirochaeta lutea* KCTC15387**	*Spirochaeta*	3,53291	1E+05	72	2910	53,8	GCA_000758165.1
Sthermo	***Spirochaeta thermophila* DSM 6578**	*Spirochaeta*	2,56022	3E+06	1	2239	60,9	GCF_000184345.1
ZAS-9	***Treponema azotonutricium* ZAS-9**	*Treponema*	3,85567	4E+06	1	3345	49,8	GCF_000214355.1
ATCC-35405	***Treponema denticola* ATCC-35405**	*Treponema*	2,826	3E+06	1	2588	37,9	GCF_000008185.1
H1-T	*Treponema denticola* H1-T	*Treponema*	2,826	3E+05	1	2588	37,9	GCF_000340605.1
Sea-81-4	*Treponema pallidum* Sea 81-4 #	*Treponema*	1,1392	1E+06	1	960	52,8	GCF_000604125.1
DSM-21527	*Turneriella parva* DSM 21527 #	*Turneriella*	4,4093	4E+06	2	4134	53,6	GCF_000266885.1

bold:Type Strain
*: No genome available for type strain
#: No type strain for the species
@ No genome available for type strain. Representative genome in NCBI

Table S3 Body sizes and wavelengths of spirochetal bacteria

Isolate	Length	Width	Wavelengths	References
Entomospira BR151	1.7 – 26.7	0.26 – 0.42	2-6	This study
Borrelia burgdorferi G25	11 – 25	0.3	2.1 – 2.4	Hovind-Hougen 1984
Borrelia burgdorferi B31	12 – 17	0.3	2.8	Hovind-Hougen 1984, Goldstein et al. 1996
Borrelia burgdorferi M6P	12.4 ± 3.7<br>(6 – 23)	0.23 ± 0.04	3.16 ± 0.8	Rudenko et al. 2016
Borrelia bissettae-like M7P	14.6 ± 3.8<br>(6 – 28)	0.32 ± 0.05	2.48 ± 0.5	Rudenko et al. 2016
Treponema hyodysenteriae	5 to 8	0.3 – 0.8		Holt 1978, najít pův citace
Treponema primitia	3 – 8	0.4		Briegel et al. 2009
Brachyspira hyodysenteriae	5.6 – 15.0	0.29 – 0.45		Sellwood R, Bland AP 1997
Leptospira barantonii	13.26 ± 2.16	0.21 ± 0.33		Thibeaux et al. 2018
Leptospira adler	11.52 ± 1.73	0.18 ± 0.17		Thibeaux et al. 2018
Leptonema illini	ND	0.15	0.702?	Goldstein et al. 1996

all measurements in µm

Supplementary Figures

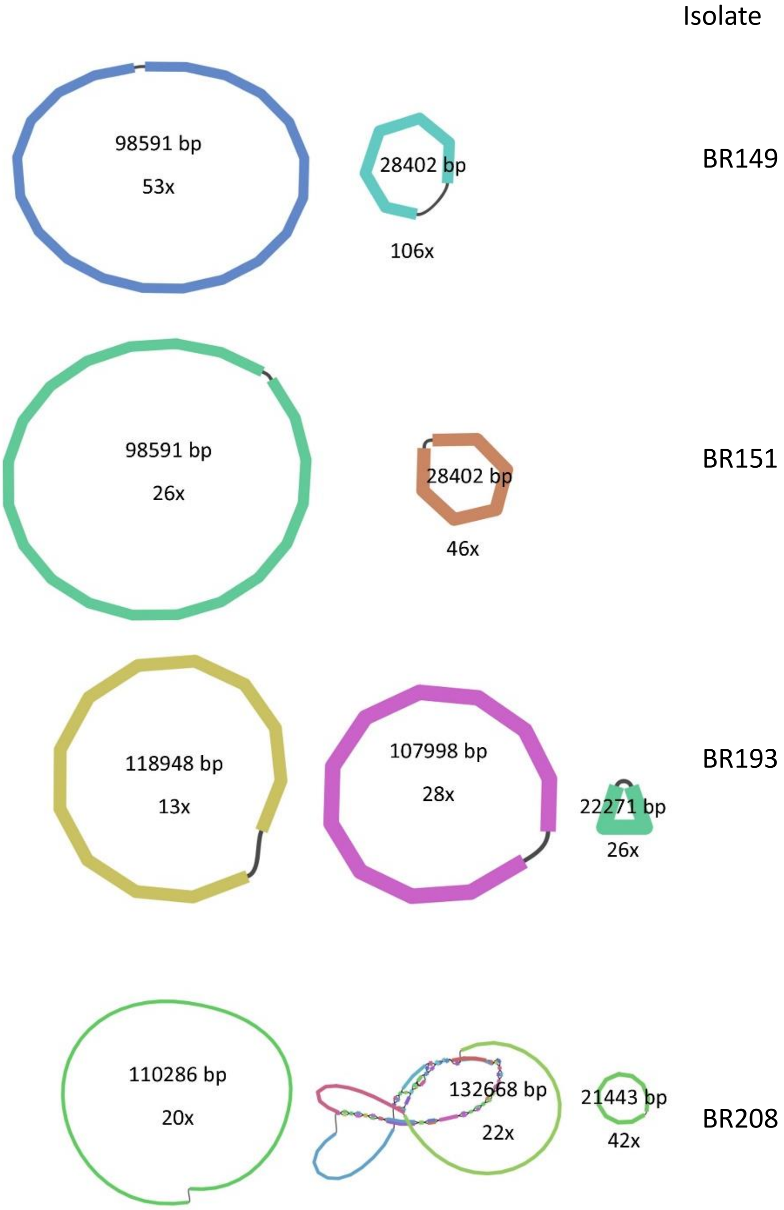

Fig S1: Visualization of plasmids found in the genus *Entomospira.* To determine the structure and size of plasmids, sequence data were analysed using the software plasmidSPAdes [1] (see Methods for details). Size (in bp) and coverage (x) are given inside or underneath the drawings. All plasmids appeared to be circular. In isolate BR193, a plasmid of appr. 8000 bp was found using SPAdes and metaSPAdes was not found by plasmidSPAdes. See text for details. Sizes are not according to scale.

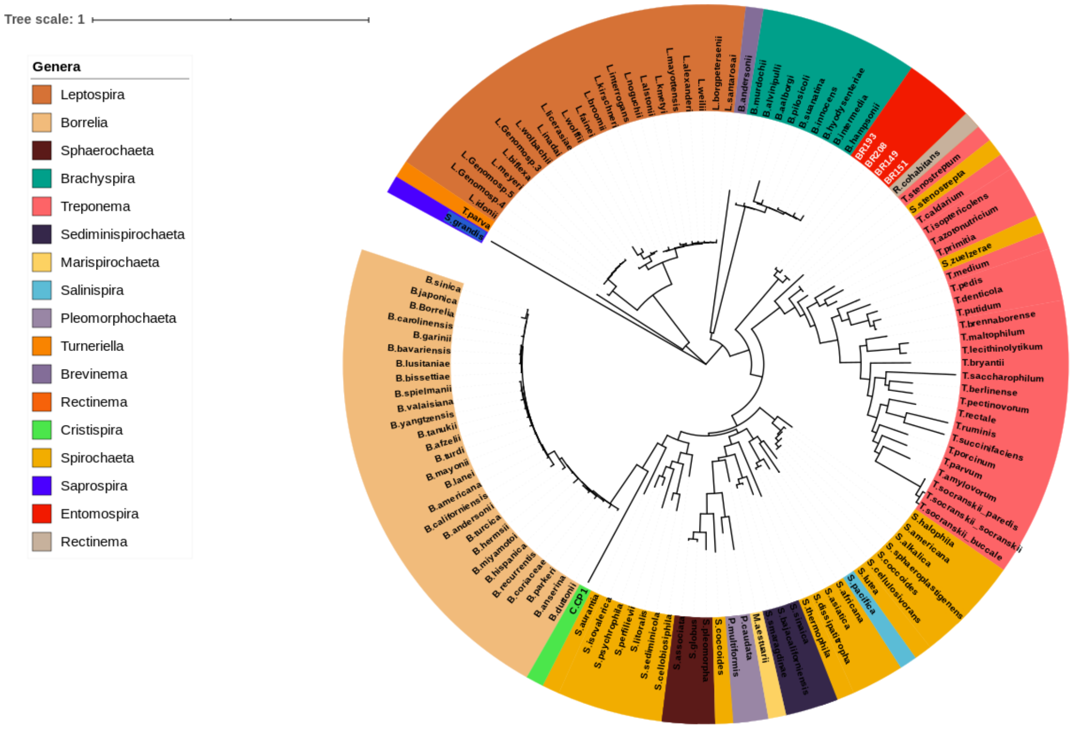

Figure S2: Maximum-likelihood phylogeny generated using RaxML based on 16S rRNA sequences [2]. A total of 16 genera of the order Spirochaetales were included (see Table S2 for details). The scale bar corresponds to substitutions per site.

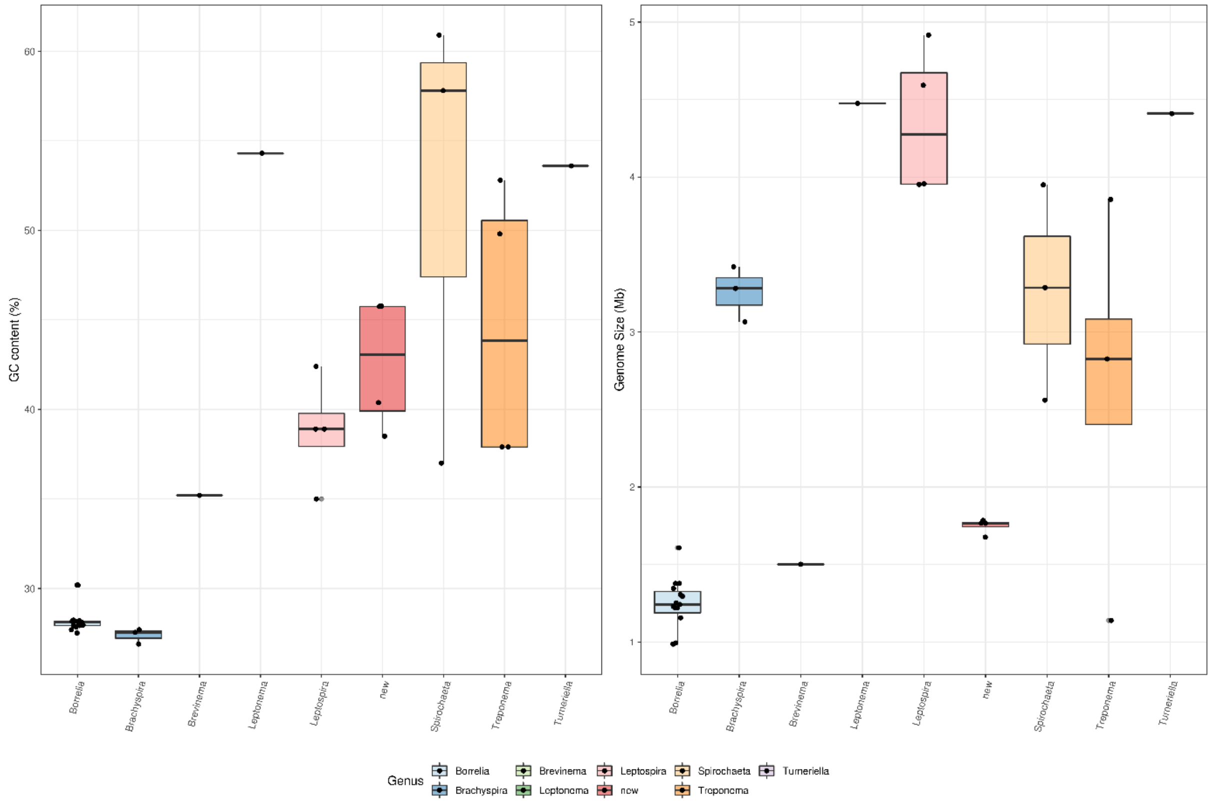

Figure S3: Variation in genome size and GC content for nine genera of the order Spirochaetales. The left hand panel shows GC content variability between genomes within genera. The highest variation in GC content is observed in the genera *Spirochaeta* and *Treponema*; Entomospira gen. nov. displays moderate variation. The right hand panel shows variation in genome size. The novel genus *Entomospira* shows very little variation in genome size compared to some other genera (e.g. *Leptospira, Spirochaeta and Treponema*).

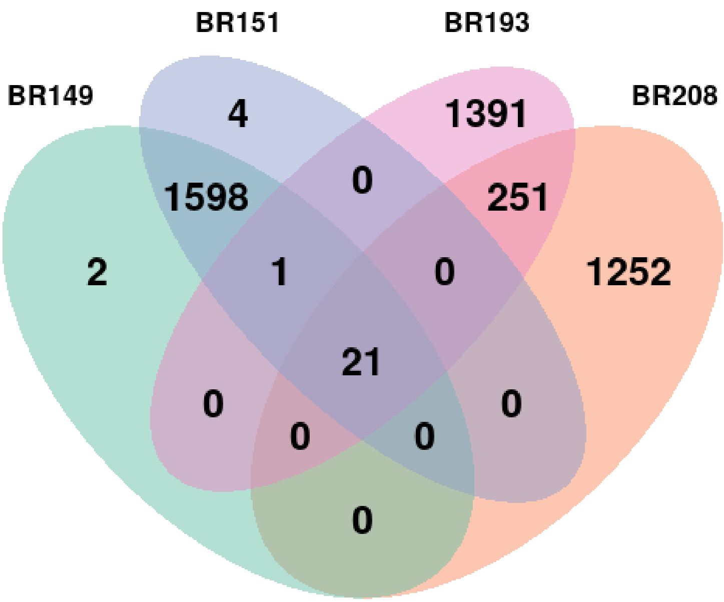

Figure S4: Venn diagram of genes shared between isolates at a level of >95 % identity. BR151 and BR149 shared 1,598 coding sequences, whilst BR193 and BR208 shared 251 genes. In BR193 and BR208 1,391 and 1,252 unique genes were found, respectively. Only 21 coding sequences were shared between all isolates.

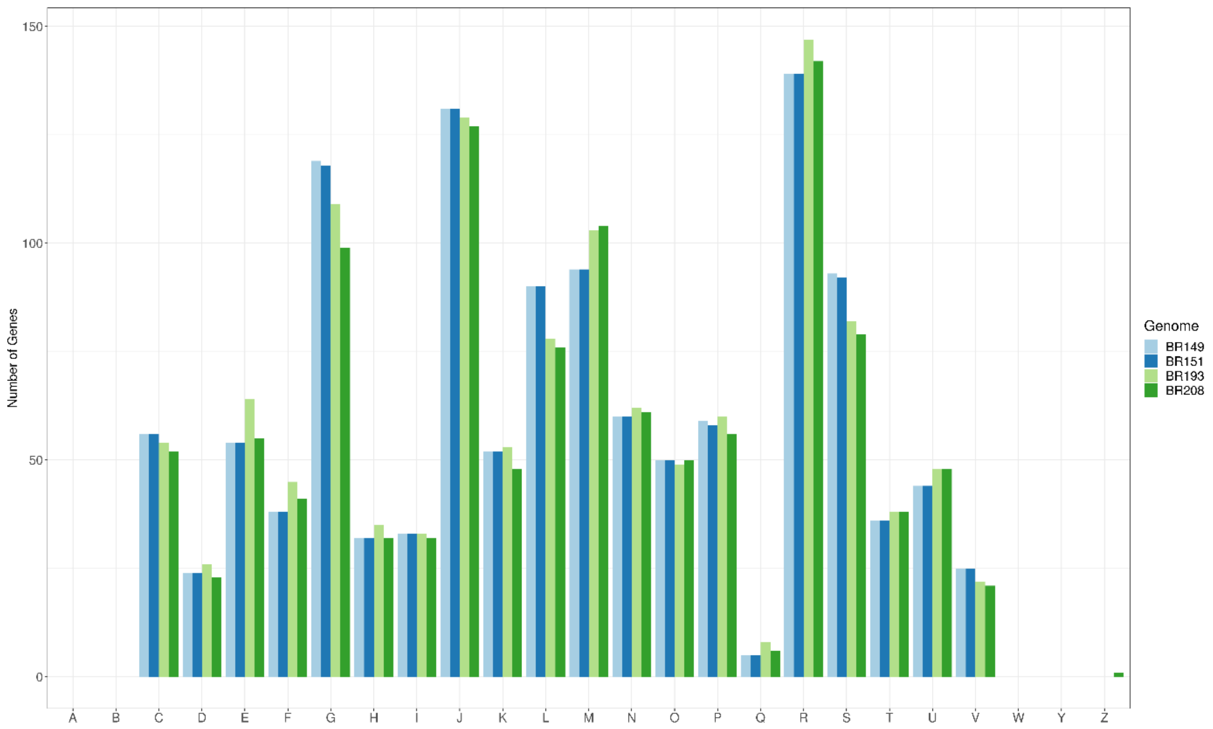

Figure S5: Analysis of functional categories (COG) between *Entomospira* species. Y-axis indicates the number of genes per category; the x-axis indicates assigned categories; the abbreviations for the functional categories are as follows: C,Energy production and conversion; D, Cell cycle control, cell division, chromosome partitioning; E, Amino acid transport and metabolism; F, Nucleotide transport and metabolism; G, Carbohydrate transport and metabolism; H, Coenzyme transport and metabolism; I, Lipid transport and metabolism; J, translation, including ribosome structure and biogenesis; K, transcription; L, replication, recombination and repair; M, cell wall structure and biogenesis and outer membrane; N, secretion, motility and chemotaxis; O, molecular chaperones and related functions; P, inorganic ion transport and metabolism; Q, Secondary metabolites biosynthesis, transport, and catabolism; R, general functional prediction only; S, no functional prediction; T, signal transduction; U, intracellular trafficking, secretion, and vesicular transport; V, defense mechanisms. The analysis showed that high proportions of genes were assigned to the categories R and S.

Supplemental movie 1. The movie shows Z-stack images of three serial tomograms joined to one volume and 3D model of a spirochete XY. Bar 200nm. For color coding see Figure4.

https://bcav-my.sharepoint.com/:v:/g/personal/vancova_paru_cas_cz/EY-3NbiczaJIg2bLFml6tvsBzHSmTO40jqEZv1Q5V9Xidg?e=1BXNLn

References:

1. Wick, R. R., Schultz, M. B., Zobel, J. & Holt, K. E. Bandage: interactive visualization of *de novo* genome assemblies. *Bioinformatics* **31**, 3350-3352 (2015).

2. Stamatakis, A. RAxML version 8: a tool for phylogenetic analysis and post-analysis of large phylogenies. *Bioinformatics* **30**, 1312-1313 (2014).
